# Pre-extensively and extensively drug-resistant tuberculosis prevalence among multidrug-resistant tuberculosis cases in Ethiopia: A systematic review and meta-analysis

**DOI:** 10.1371/journal.pntd.0014744

**Published:** 2026-09-28

**Authors:** Muluneh Assefa, Mitkie Tigabie, Abebaw Setegn, Yenesew Mihret Wondmagegn, Azanaw Amare, Getu Girmay, Sirak Biset, Wesam Taher Almagharbeh

**Affiliations:** 1 Department of Medical Microbiology, School of Biomedical and Laboratory Sciences, College of Medicine and Health Sciences, University of Gondar, Gondar, Ethiopia; 2 Department of Medical Parasitology, School of Biomedical and Laboratory Sciences, College of Medicine and Health Sciences, University of Gondar, Gondar, Ethiopia; 3 Department of Immunology and Molecular Biology, School of Biomedical and Laboratory Sciences, College of Medicine and Health Sciences, University of Gondar, Gondar, Ethiopia; 4 Department of Medical and Surgical Nursing, Faculty of Nursing, University of Tabuk, Tabuk, Saudi Arabia; Shanghai Jiao Tong University, CHINA

## Abstract

**Background:**

The burden of pre-extensively drug-resistant tuberculosis (Pre-XDR-TB) and extensively drug-resistant tuberculosis (XDR-TB) cases in Ethiopia has become one of the most challenging problems for monitoring the treatment of patients with multidrug-resistant tuberculosis (MDR-TB). This study aimed to provide a valuable evidence synthesis on the pooled prevalence of Pre-XDR and XDR-TB among MDR-TB patients in Ethiopia.

**Methodology:**

This study was conducted in accordance with the Preferred Reporting Items for Systematic Reviews and Meta-Analyses (PRISMA) guidelines. Extracted data from relevant articles were analyzed using STATA version 17.0. The pooled estimate of the effect size was computed using a restricted maximum likelihood random-effects model, considering a 95% confidence interval, and a forest plot was used to visualize the proportion of Pre-XDR and XDR-TB. Because the Pre-XDR and XDR-TB cases are rare, the Freeman-Tukey double arcsine transformation was applied to estimate the pooled proportions. The I2 statistic and Galbraith plot confirmed heterogeneity. A univariate meta-regression, sensitivity, and subgroup analyses were conducted to identify the source of heterogeneity. Egger’s test and funnel plot were used to check publication bias.

**Results:**

Eight studies comprising 1,518 MDR-TB patients, with 656 new and 862 previously treated cases, were included. Among those, 52 Pre-XDR and 13 XDR-TB cases were identified. According to the Freeman-Tukey transformation, the overall pooled prevalence of Pre-XDR-TB was 3% (95% CI: 2%-5%, I2 = 43.69%) and XDR-TB was 1% (95% CI: 0%-4%, I2 = 54.07%). In the subgroup analysis, the prevalence of Pre-XDR-TB across studies from multiple regions of Ethiopia was 7% (95% CI: 3%-13%), and in Addis Ababa, it was 3% (95% CI: 2%-4%). Single studies in the Amhara, Tigray, and Oromia regions reported Pre-XDR-TB prevalence of 6%, 5%, and 3%, respectively. The pooled prevalence of XDR-TB in Addis Ababa was 1% (95% CI: 0%-2%). A single study conducted in Oromia reported a relatively higher prevalence of XDR-TB; 10% (95% CI: 3%-26%). Regarding the study year, the XDR-TB prevalence was 3% (95% CI: 0%-11%) in 2005–2018 and 1% (95% CI: 0%-2%) in 2019–2024. A statistically significant publication bias was observed in XDR-TB prevalence (p = 0.024).

**Conclusion:**

There is a significant prevalence of Pre-XDR and XDR-TB cases among MDR-TB patients in Ethiopia. It highlights the need for strengthened surveillance in all regions and expanded drug susceptibility testing of local strains for better management of MDR-TB patients.

## Author summary

The emergence of Pre-XDR and XDR-TB poses a severe threat to public health in Ethiopia, complicating patient monitoring and treatment success. This study aimed to synthesize evidence on the pooled prevalence of these highly resistant strains among MDR-TB patients across various Ethiopian regions. We conducted a systematic review and meta-analysis of eight studies involving 1,518 MDR-TB patients using PRISMA guidelines. The pooled prevalence analysis was calculated based on the study’s sample size, which reported 52 Pre-XDR-TB cases from 1,472 MDR-TB patients and 13 XDR-TB cases from 836 MDR-TB patients, separately. Our Freeman-Tukey transformed meta-analysis revealed a pooled prevalence of 3% for Pre-XDR-TB and 1% for XDR-TB among the reported MDR-TB patients. Subgroup analysis showed geographic variation, with a pooled prevalence of XDR-TB ranging from 1% in Addis Ababa to 10% in the Oromia region. Although temporal trends indicated a relatively higher prevalence in earlier study periods (2005–2018) compared to recent years (2019–2024), studies differ in case definitions, drug-susceptibility testing panels, diagnostic methods, populations, and geographic coverage, and are affected by the small number and heterogeneity of the included studies. The COVID-19 pandemic also had an impact on the under-detection of drug-resistant TB cases. Moreover, recent data revealed critical resistance to newer Group A drugs, including emerging dual resistance to fluoroquinolones combined with bedaquiline or linezolid among MDR-TB isolates.The results indicate a significant prevalence of highly drug-resistant TB within Ethiopian MDR-TB treatment centers. These findings highlight an urgent need for the national TB program to strengthen laboratory surveillance, expand drug susceptibility testing, and enhance resource allocation for managing complex cases. Standardizing diagnostic tools across all regions is essential to close existing surveillance gaps and improve treatment outcomes for drug-resistant TB.

## Background

Ethiopia suffers from a significant burden of tuberculosis (TB), and the emergence of drug-resistant Mycobacterium tuberculosis (MTB) strains poses a serious threat to public health [1]. Among these, pre-extensively drug-resistant tuberculosis (Pre-XDR-TB) and extensively drug-resistant tuberculosis (XDR-TB) cases represent the most challenging issues for the treatment and monitoring of multidrug-resistant tuberculosis (MDR-TB) patients. An estimated 140 new TB cases and 19 TB-related fatalities per 100,000 people are reported in Ethiopia each year, making it one of the 30 nations with the highest TB and TB/human immunodeficiency virus burden across the world [2].

According to a systematic review and meta-analysis report in Ethiopia, unemployment, previous TB history, contact with a known MDR-TB patient, and having pulmonary TB were the risk factors of drug-resistant TB. In addition, a previous history of TB treatment is a major risk factor for acquiring drug-resistant TB in Ethiopia, which might be due to poor adherence during the first-line anti-TB treatment [3]. Several factors associated with the occurrence of Pre-XDR and XDR-TB include the previous history of TB treatment and its duration, rates of treatment failure, HIV co-infection among drug-resistant TB patients, geographical variations within Ethiopia, differences in TB control programs and drug resistance patterns, and methodologies used for drug susceptibility testing across different studies [4,5].

This comprehensive meta-analysis pooled data from various Ethiopian studies reporting drug susceptibility testing among MTB isolates. The review encompassed studies utilizing phenotypic and genotypic methods to detect resistance to first-line drugs (defining MDR-TB), first-line drugs and fluoroquinolones (a key characteristic of Pre-XDR-TB), and first-line drugs, fluoroquinolones, and second-line injectable agents or Group A drugs (defining XDR-TB). Although there are previous systematic review and meta-analysis reports on the burden of MDR-TB and rifampicin-resistant TB in Ethiopia [6–8], this study is the first to determine the magnitude of Pre-XDR and XDR-TB epidemics in the county.

Accurate prevalence estimates are essential for guiding the development and implementation of effective national TB control strategies, allocating resources for the diagnosis and management of highly drug-resistant TB, monitoring trends in drug resistance over time, identifying high-risk populations, and geographical areas for targeted interventions. Therefore, this study would provide a significant summary data on the prevalence of Pre-XDR and XDR-TB cases among the MDR-TB population in Ethiopia, contributing significantly to the knowledge base and informing public health responses to these challenging strains of MTB.

This study aimed to provide a valuable evidence synthesis on the pooled prevalence of Pre-XDR and XDR-TB among MDR-TB patients in Ethiopia. We hypothesized that the prevalence of these highly resistant strains would show significant variations across different geographical regions of the country, reflecting differences in regional TB control programs, and would exhibit a temporal trend over the last two decades. Furthermore, we anticipated that the type of diagnostic methodology (phenotypic versus genotypic) would influence the reported prevalence rates.

## Methodology

### Study design and reporting

The Preferred Reporting Items for Systematic Reviews and Meta-Analyses (PRISMA) guidelines were used to report the findings [9] (S1 Checklist). The protocol for this systematic review and meta-analysis was pre-registered with the International Prospective Register of Systematic Reviews (PROSPERO) under registration number CRD42024536643. We confirm that the study was conducted in full alignment with the registered protocol, with relevant deviations on the statistical analysis plan that used the Freeman-Tukey double arcsine transformation method to provide a correct comparison of the pooled estimate proportion for the rare event (S1 Table).

### Definition of terms

For this review, we adopted the most recent World Health Organization (WHO) definitions (2024). However, recognizing that the definitions of XDR-TB evolved during the study period (2005–2024), we extracted prevalence data from earlier studies using the definitions in effect at the time of their conduct. This operational heterogeneity is addressed through subgroup analysis by study period.

- Multidrug-resistant tuberculosis (MDR-TB): it is the resistance of MTB to rifampicin and isoniazid drugs [10].
- Pre-extensively drug-resistant tuberculosis (Pre-XDR-TB): refers to MDR-TB with resistance to any fluoroquinolone [11].
- Extensively drug-resistant tuberculosis (XDR-TB): defined as MDR-TB with additional resistance to any fluoroquinolone and at least one Group A drug [11].

### Research question and framework

The primary research question was: What is the pooled prevalence of Pre-XDR and XDR-TB among MDR-TB patients in Ethiopia? To address this, we followed the COCOPOP framework:

- Condition (CO): Pre-extensively drug-resistant (Pre-XDR) and extensively drug-resistant (XDR) tuberculosis.
- Context (CO): All regions and healthcare settings within Ethiopia.
- Population (POP): Patients diagnosed with multidrug-resistant tuberculosis (MDR-TB).

### Literature search strategy

The search included all published studies, and the final search was conducted between November 01 and 10, 2025. The following electronic databases were used: PubMed/Medline, Scopus, EMBASE, Google Scholar, and Cochrane Library to identify articles reporting the prevalence of Pre-XDR and XDR-TB among MDR-TB cases in Ethiopia. We used search terms alone or in combination with Boolean operators such as “OR” or “AND”. The PubMed search strategy was as follows: (((((pre-extensively drug-resistant tuberculosis) OR (extensively drug-resistant tuberculosis)) OR (Pre-XDR-TB)) OR (XDR-TB)) AND ((multidrug-resistant tuberculosis) OR (MDR-TB))) AND (Ethiopia). The search terms applied in each database are provided in S2 Table. A manual Google search of gray literature and a systematic search of their respective references were conducted. The retrieved articles were imported into the EndNote X9 bibliographic software manager (Clarivate Analytics, Philadelphia, PA).

### Study selection and eligibility criteria

Five authors (MA, AS, YMW, GG, and AA) independently screened the titles and abstracts of the articles. The articles were then assessed for eligibility, and any disagreements between the authors were resolved through discussion. This study included English-language articles (as no studies were published in other languages), with no time-period limit, observational studies, and studies reporting Pre-XDR and XDR-TB among MDR-TB patients. Studies were excluded if they met any of the following criteria: (1) case reports, communications, letters to editors, opinions, reviews, or meta-analyses; (2) studies conducted on populations other than TB patients; or (3) studies with incomplete or non-standardized reporting that prevented accurate data extraction. Specifically, we excluded studies that did not report a clear denominator (the total number of MDR-TB patients). This rigorous selection process was maintained to minimize outcome reporting bias and ensure the validity of the pooled prevalence estimates.

### Quality assessment of the studies

Articles were retrieved for review, and relevant information was carefully extracted. The Joana Briggs Institute (JBI) critical appraisal checklist for simple prevalence was used to assess the quality of included studies [12]. Articles of high and medium quality were included in this systematic review and meta-analysis (S3 Table). Four independent authors (MA, MT, AA, and GG) assessed the quality of the included studies.

### Outcome of interest

The main outcome of the study was to determine the pooled prevalence of Pre-XDR and XDR-TB among MDR-TB cases in Ethiopia.

### Data extraction

Data from individual studies were extracted by five independent authors (MA, AS, MT, AA, and SB) using Microsoft Excel. The information collected from the eligible studies included the authors, year of publication, study area, study design, detection method of drug resistance, number of Pre-XDR-TB cases, number of XDR-TB cases, and number of MDR-TB cases.

### Data analysis

Extracted data were exported to STATA version 17.0 for analysis. Subgroup analysis was performed to evaluate differences based on study area, study year, and detection method of drug resistance. The pooled prevalence of Pre-XDR and XDR-TB and their respective 95% confidence intervals are visually displayed using a forest plot. Pooled estimates are presented as proportions with a Freeman-Tukey double arcsine transformation to ensure that all confidence intervals are mathematically bounded between 0 and 1, thereby avoiding implausible negative estimates. While the forest plot displays these values on a unit scale (0–1) for statistical consistency, all results are reported as percentages in the text for clinical interpretability. Heterogeneity between the studies was evaluated using the Galbraith plot and an index of heterogeneity (I2 statistic) value of 0% = no heterogeneity, ≤ 25% = low, 25%–50% = moderate, 50–75 = substantial, and ≥ 75% = high [13]. In all pooled analyses, heterogeneity resulting from differences in effects across studies was determined using a random-effects model. A sensitivity analysis of the effect of each study on overall prevalence was also conducted. Publication bias was statistically investigated using Egger’s test [14] and visual inspection of funnel plots. A p-value of less than 0.05 in Egger’s test was considered to indicate statistically significant publication bias. To investigate potential sources of heterogeneity and small-study effects, a univariate meta-regression was conducted. The study sample size was selected as a covariate to evaluate whether the size of the study population influenced the reported prevalence, which can serve as an indicator of methodological variation between large-scale surveillance and small-center studies. Publication year was also included to test for temporal trends in drug resistance. The confirmatory analysis was the pooled estimate for Pre-XDR and XDR-TB using a random-effects model. Exploratory analyses were the sources of heterogeneity, subgroup analyses, and meta-regressions. These were pre-specified to test the secondary hypotheses that prevalence rates significantly differ based on geographical location, study period (comparing early 2005–2018 data to more recent 2019–2024 data), and detection method (phenotypic and genotypic drug susceptibility testing). The results are presented in the tables, text, and figures.

## Results

### Search results

Initially, 433 potentially relevant articles were identified from different databases. After excluding duplicates, 81 articles were screened. Then, the full text of 15 possibly related articles was assessed for eligibility. After removing 7 articles, which were not original studies, 8articles were used for the final systematic review and meta-analysis [1,15–21] (Fig 1).

**Fig 1:**
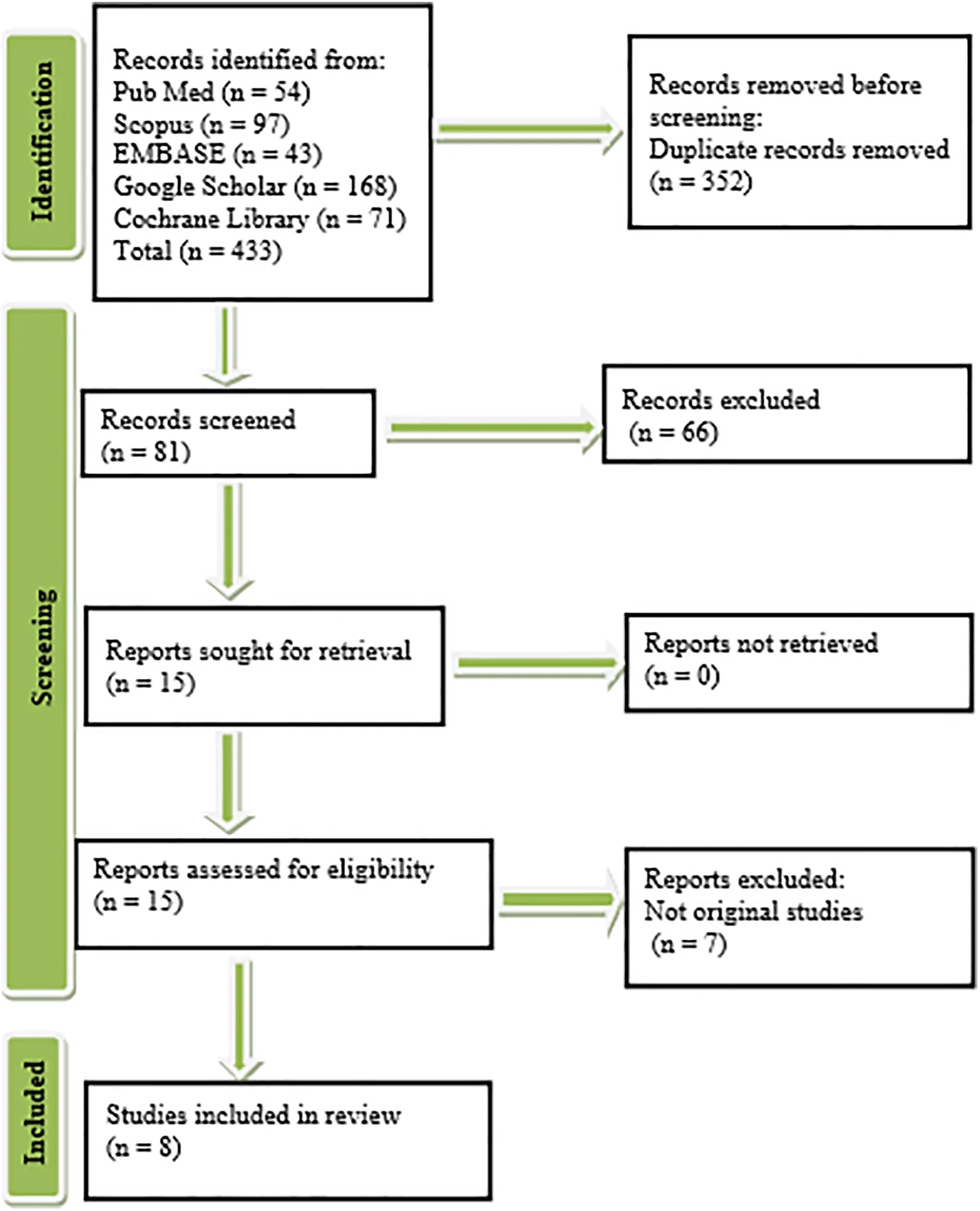
Flow diagram describing the selection of studies for the systematic review and meta-analysis.

### Study characteristics

Among the eight studies included in this review, 3 studies were conducted in Addis Ababa, followed by multiple regions (n = 2), with one study each for Tigray, Amhara, and Oromia regions. All the studies used a cross-sectional study design. Five studies used a genotypic detection method for TB drug resistance, while three studies used phenotypic drug susceptibility testing (Table 1).

**Table 1:** Characteristics of studies reported the prevalence of Pre-XDR and XDR-TB among MDR-TB cases in Ethiopia.

Author (reference)	Study area	Study period	Study design	Detection method for drug resistance	Sample size (MDR-TB)			Pre-XDR-TB			XDR-TB		
					Total	New cases	Previously treated cases	Total	New cases	Previously treated cases	Total	New cases	Previously treated cases
Diriba et al, 2025 [15]	Addis Ababa	2022-24	Cross-sectional	Phenotypic	468	262	206	11	4	7	5	3	2
Diriba et al, 2022 [16]	Addis Ababa	2019-22	Cross-sectional	Genotypic	644	261	383	19	5	14	–	–	–
Dagne et al, 2021 [17]	Multiple regions*	2019-20	Cross-sectional	Genotypic	99	37	62	8	1	7	1	–	1
Yenew et al, 2024 [1]	Multiple regions**	2019-20	Cross-sectional	Phenotypic	19	3	16	1	–	–	1	–	1
Welekidan et al, 2020 [18]	Tigray region	2018-19	Cross-sectional	Genotypic	38	20	18	2	–	2	–	–	–
Bedru et al, 2021 [20]	Oromia region	2017-18	Cross-sectional	Genotypic	30	8	22	1	–	–	3	–	–
Shibabaw et al, 2020 [19]	Amhara region	2016-18	Cross-sectional	Genotypic	174	64	110	10	1	9	1	1	–
Agonafir et al, 2010 [21]	Addis Ababa	2005-6	Cross-sectional	Phenotypic	46	1	45	–	–	–	2	–	–
Total					1518	656	862	52	11	39	13	4	4

Note. MDR-TB; Multidrug-resistant tuberculosis, Pre-XDR-TB; pre-extensively drug-resistant tuberculosis, XDR-TB; extensively drug-resistant tuberculosis, Multiple regions*; St. Peter TB Specialized Hospital, Shambu Hospital, Gambella Hospital, Tulu Bolo Hospital, Ambo Hospital, Fitche Hospital, Asosa Hospital, and Yirgalem Hospital, Multiple regions**; Amhara, Gambella, and Benishangul-Gumuz, Not reported (-)

### The pooled prevalence of Pre-XDR and XDR-TB cases

A total of eight studies [1,15–21] comprising 1,518 MDR-TB patients, with 656 new and 862 previously treated cases included. The first single new MDR-TB case was reported by Agonafir et al. in 2010, and the highest case was reported by Diriba et al., 2025. From these, 52 were Pre-XDR and 13 were XDR-TB cases. For the accurate estimation, the pooled prevalence analysis involved two distinct categories based on their respective sample size: the Pre-XDR-TB comprised a total sample size of 1,472 MDR-TB patients in seven studies by excluding a sample size of a study not reported Pre-XDR-TB by Agonafir et al, 2010, while the XDR-TB prevalence was calculated from 836 MDR-TB patients by excluding a sample size of studies by Diriba et al., 2022 and Welekidan et al., 2020, which did not report XDR-TB, as presented in Table 1. According to the Freeman-Tukey transformed estimate, the pooled prevalence of Pre-XDR-TB was 3% (95% CI: 2%-5%, I2 = 43.69) (Fig 2) and XDR-TB was 1% (95% CI: 0%-4%, I2 = 54.07) (Fig 3). In the heterogeneity analysis, there was moderate variation between the studies reporting Pre-XDR-TB (Fig 4), with substantial heterogeneity observed in studies on XDR-TB prevalence, as shown in the Galbraith plot (Fig 5).

**Fig 2:**
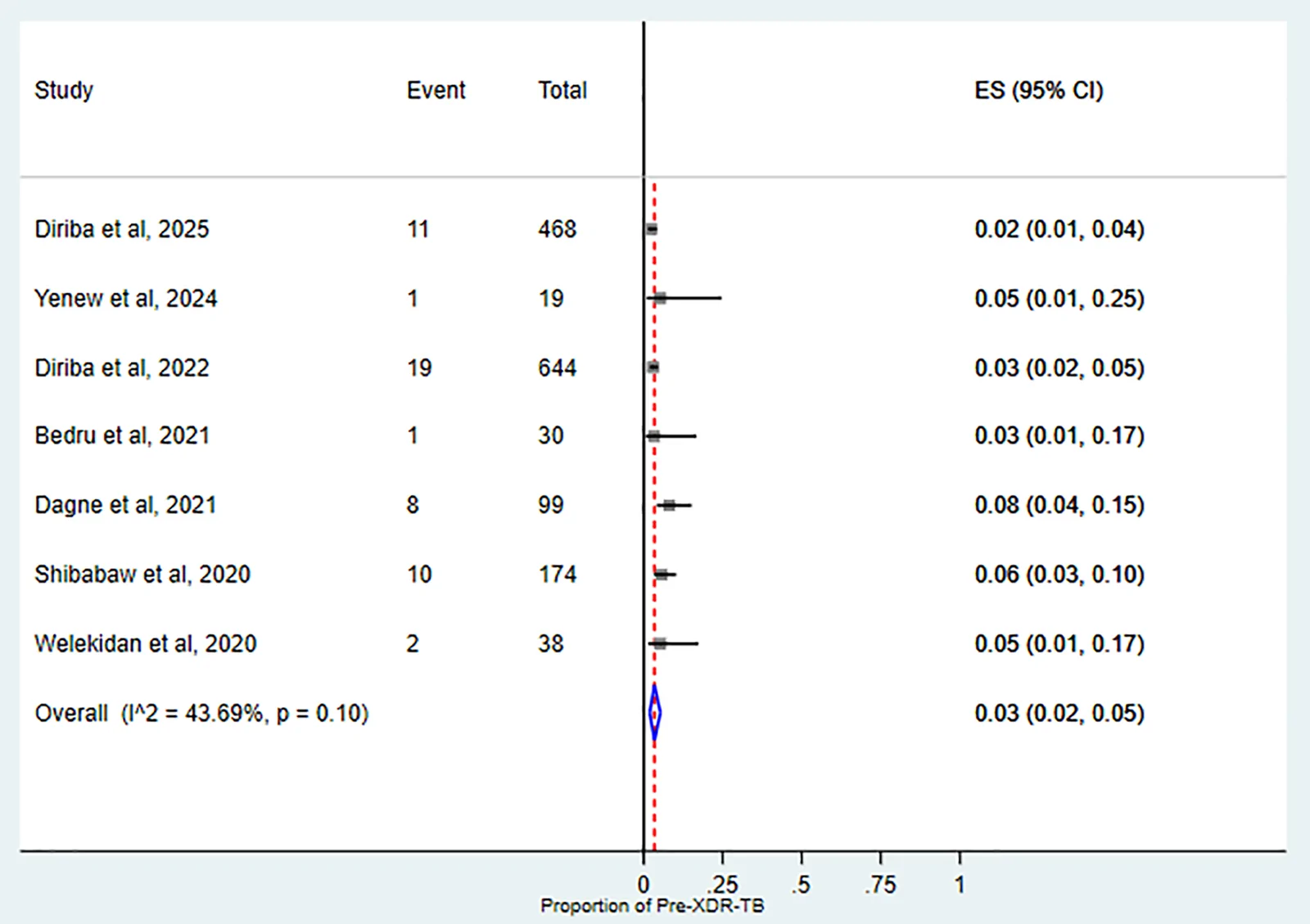
Forest plot showed the pooled prevalence of Pre-XDR-TB cases.

**Fig 3:**
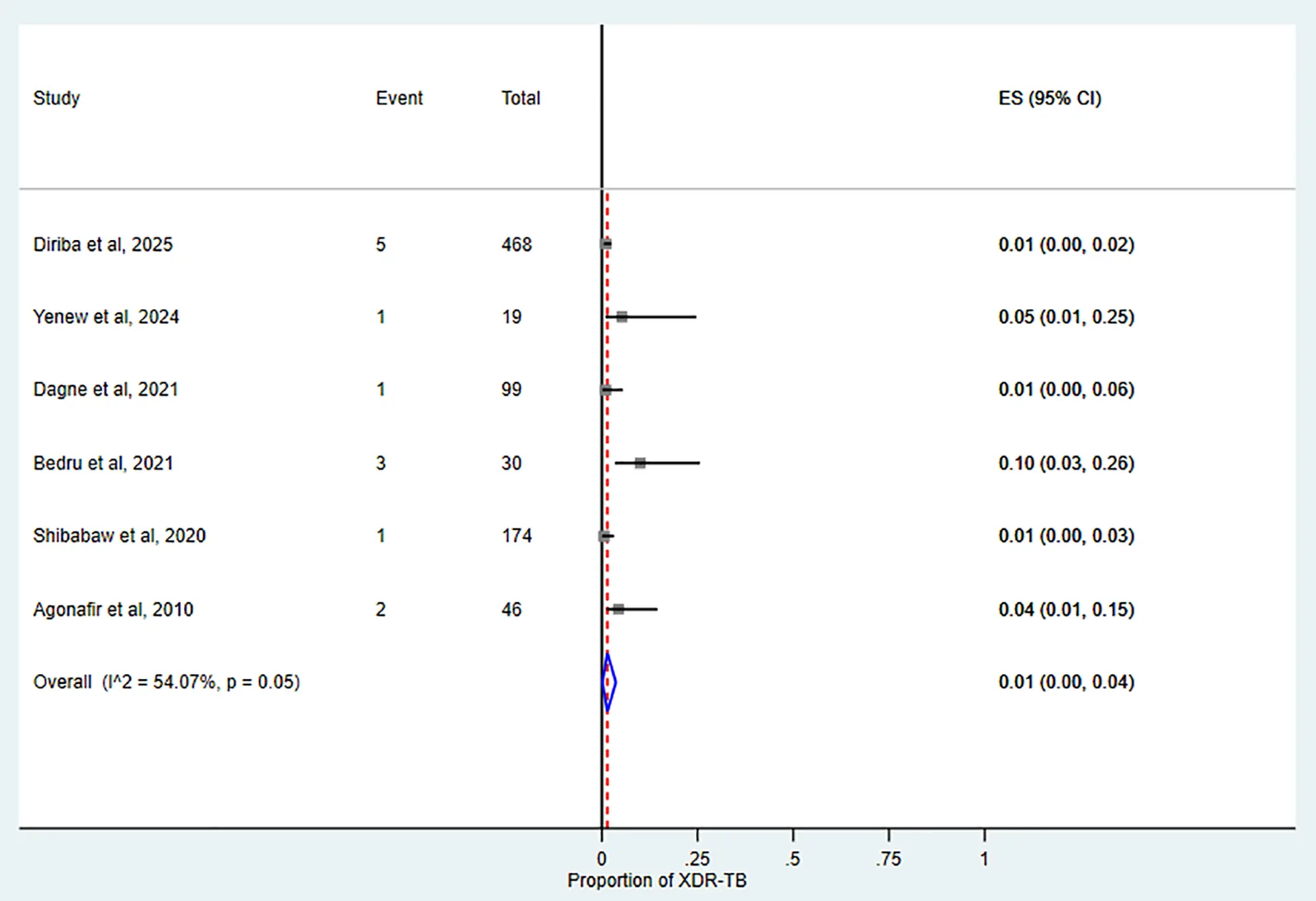
The forest plot showed the pooled prevalence of XDR-TB cases.

**Fig 4:**
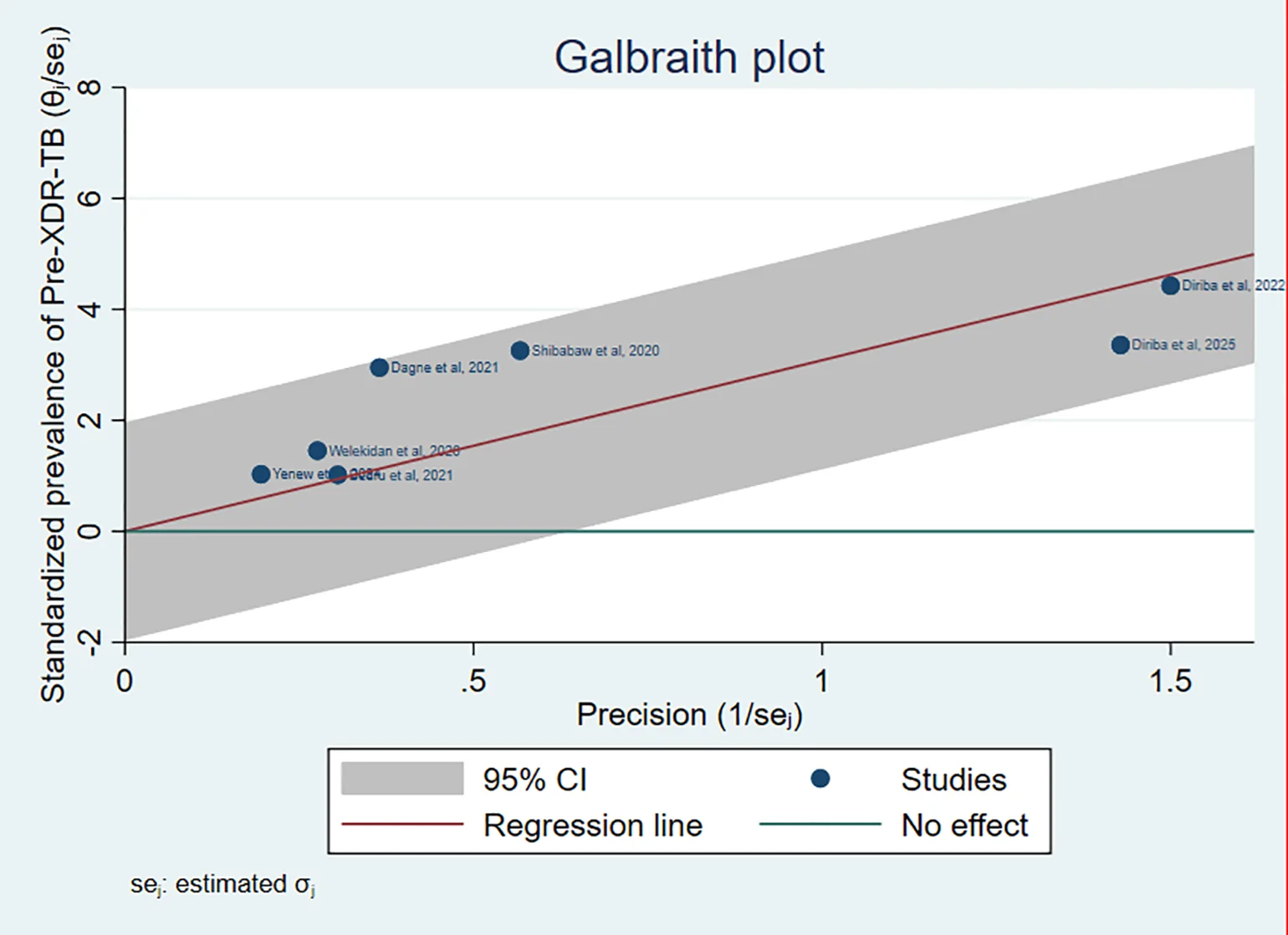
Galbraith plot for heterogeneity of Pre-XDR-TB prevalence.

**Fig 5:**
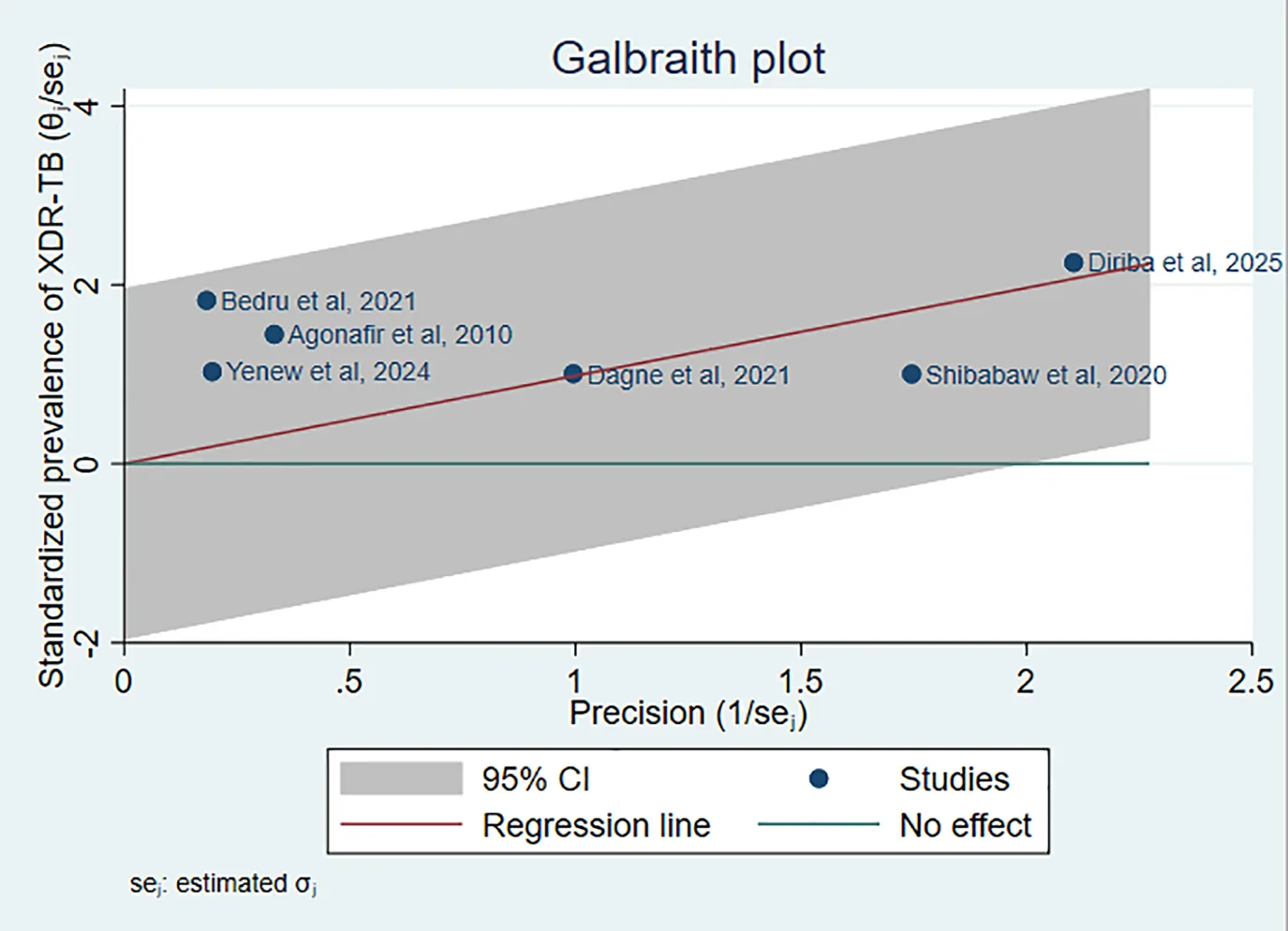
Galbraith plot for heterogeneity of XDR-TB prevalence.

### Subgroup analysis

A subgroup analysis was conducted to identify the source of variation. A relatively higher prevalence of Pre-XDR-TB was observed in studies in multiple regions of Ethiopia (7%; 95% CI: 3%-13%), followed by Addis Ababa (3%; 95% CI: 2%-4%). Single studies in the Amhara, Tigray, and Oromia regions reported Pre-XDR-TB prevalence of 6%, 5%, and 3%, respectively (Fig 6). Based on the detection method of drug resistance, the prevalence of Pre-XDR-TB among the studies that used genotypic and phenotypic methods was 4% and 1%, respectively (Fig 7). The substantial heterogeneity shown between studies conducted on XDR-TB prevalence might be due to variations in geographic location and study year. Based on the study area, the pooled prevalence of XDR-TB in two studies conducted in Addis Ababa was 1% (95% CI: 0%-2%). A single study conducted in Oromia reported an XDR-TB prevalence of 10% (95% CI: 3%-26%) (Fig 8). According to the study year, the XDR-TB prevalence was 3% (95% CI: 0%-11%) in 2005–2018 and 1% (95% CI: 0%-2%) in 2019–2024 (Fig 9). Based on the detection method of drug resistance, the prevalence of XDR-TB among the studies that used phenotypic and genotypic methods showed no difference, which was 2% each (Fig 10). While variations were observed across different regions and time periods, these findings should be interpreted with caution. For instance, estimates for certain regions were derived from single studies, and the resulting wide confidence intervals suggest substantial statistical instability. Rather than definitive national trends, these data provide a preliminary indication of geographic variation in drug resistance patterns.

**Fig 6:**
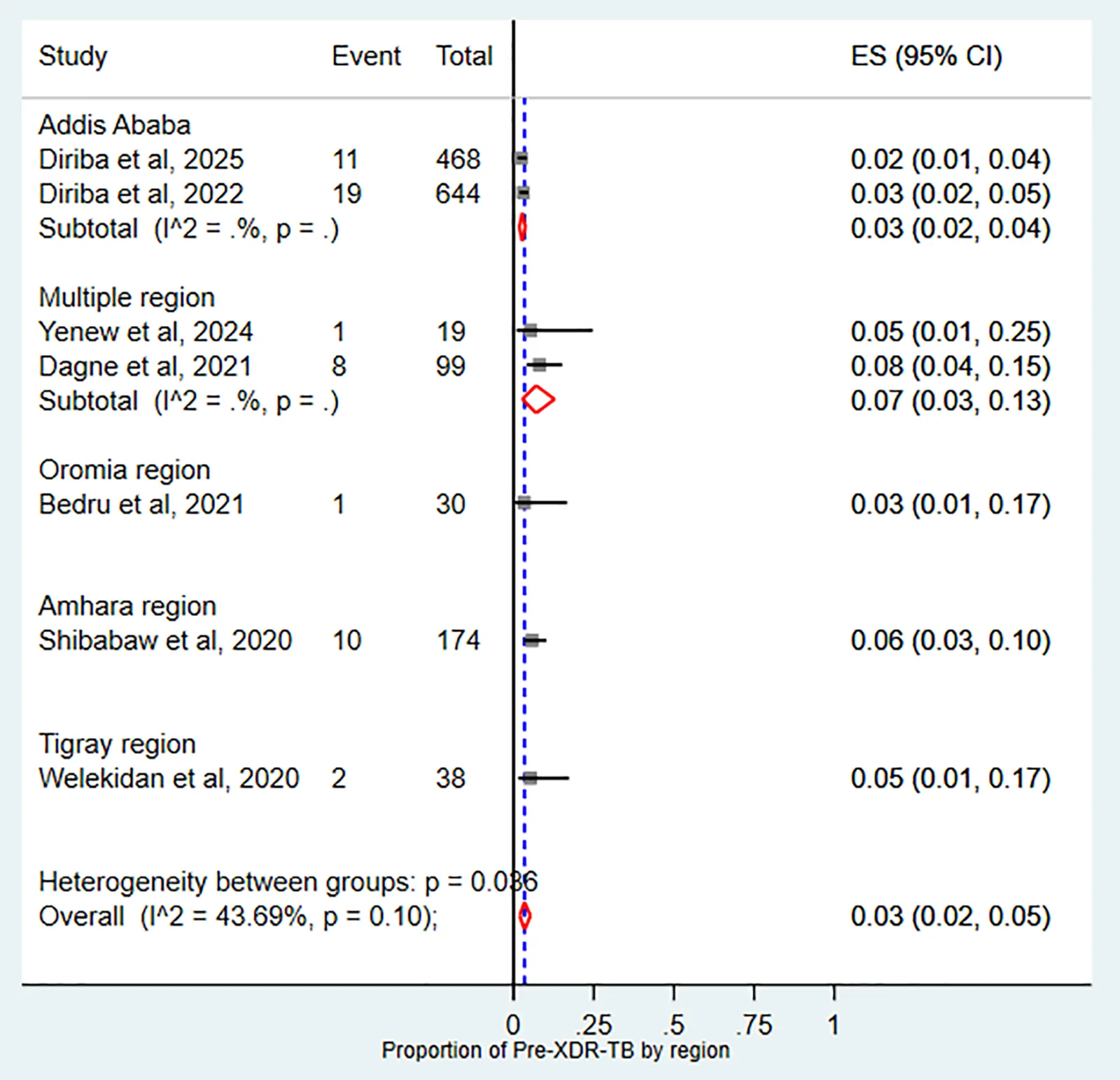
The forest plot showed the Pre-XDR-TB subgroup analysis based on the study area.

**Fig 7:**
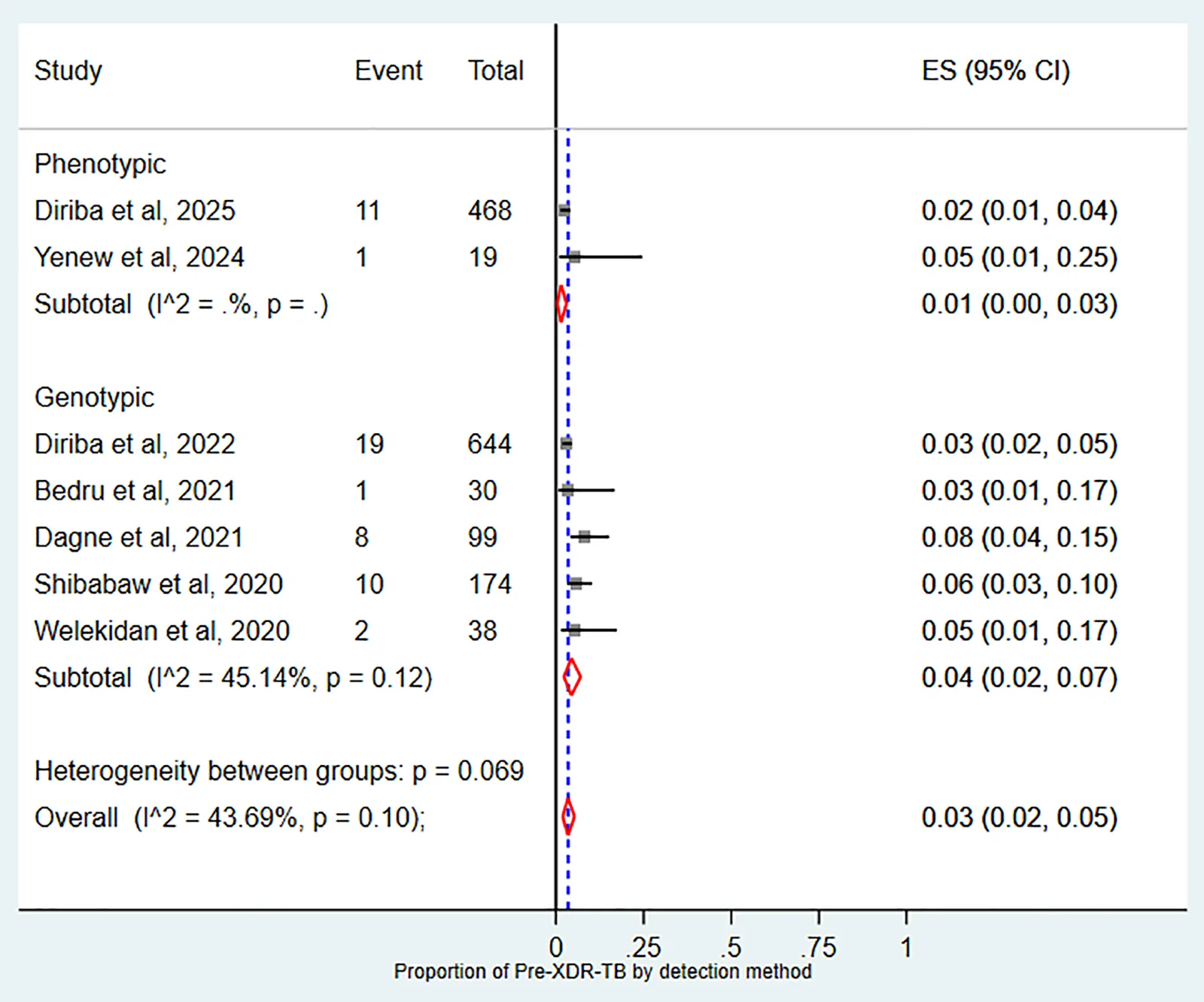
The forest plot showed the Pre-XDR-TB subgroup analysis based on the detection method.

**Fig 8:**
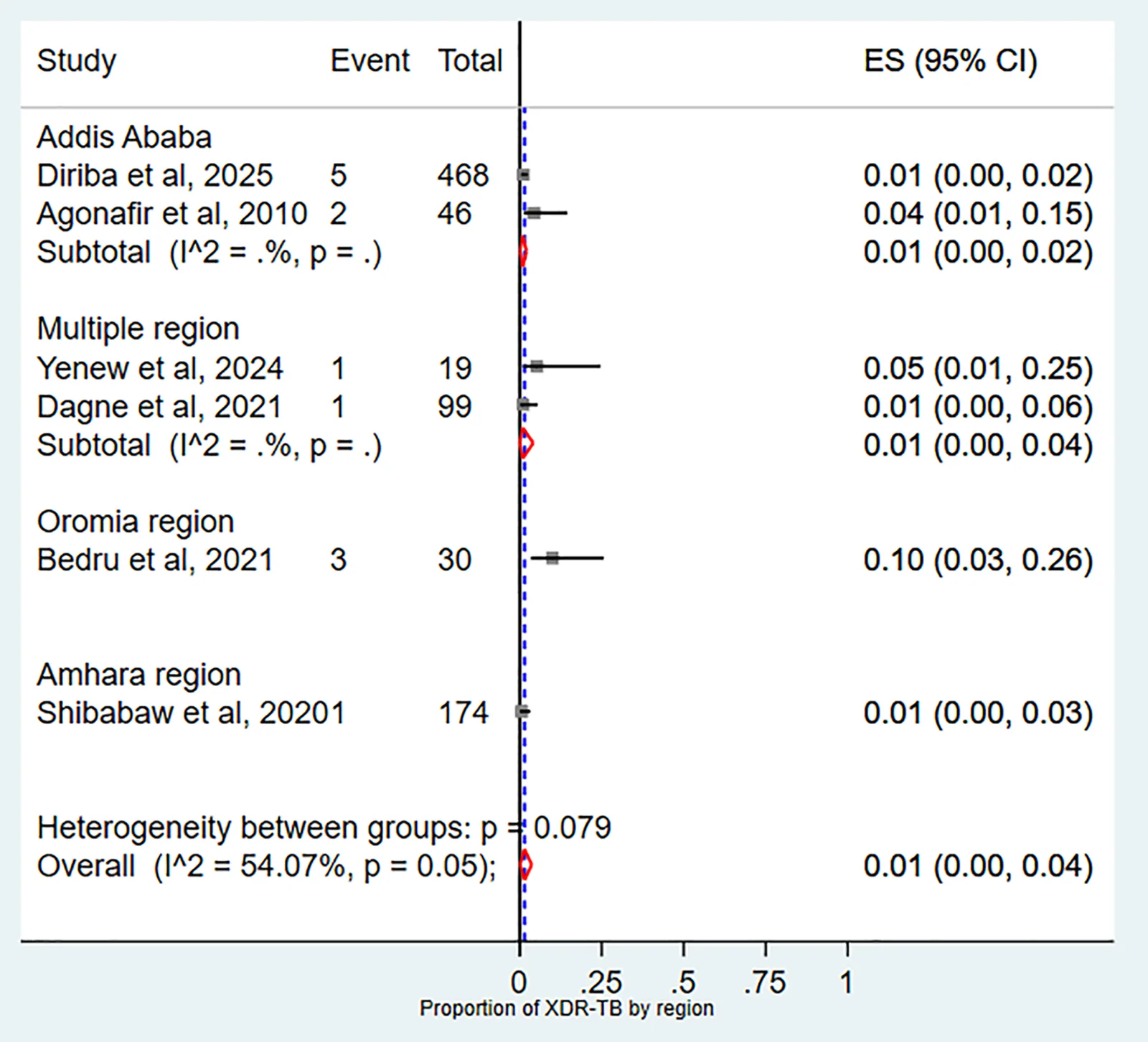
The forest plot showed XDR-TB subgroup analysis based on the study area.

**Fig 9:**
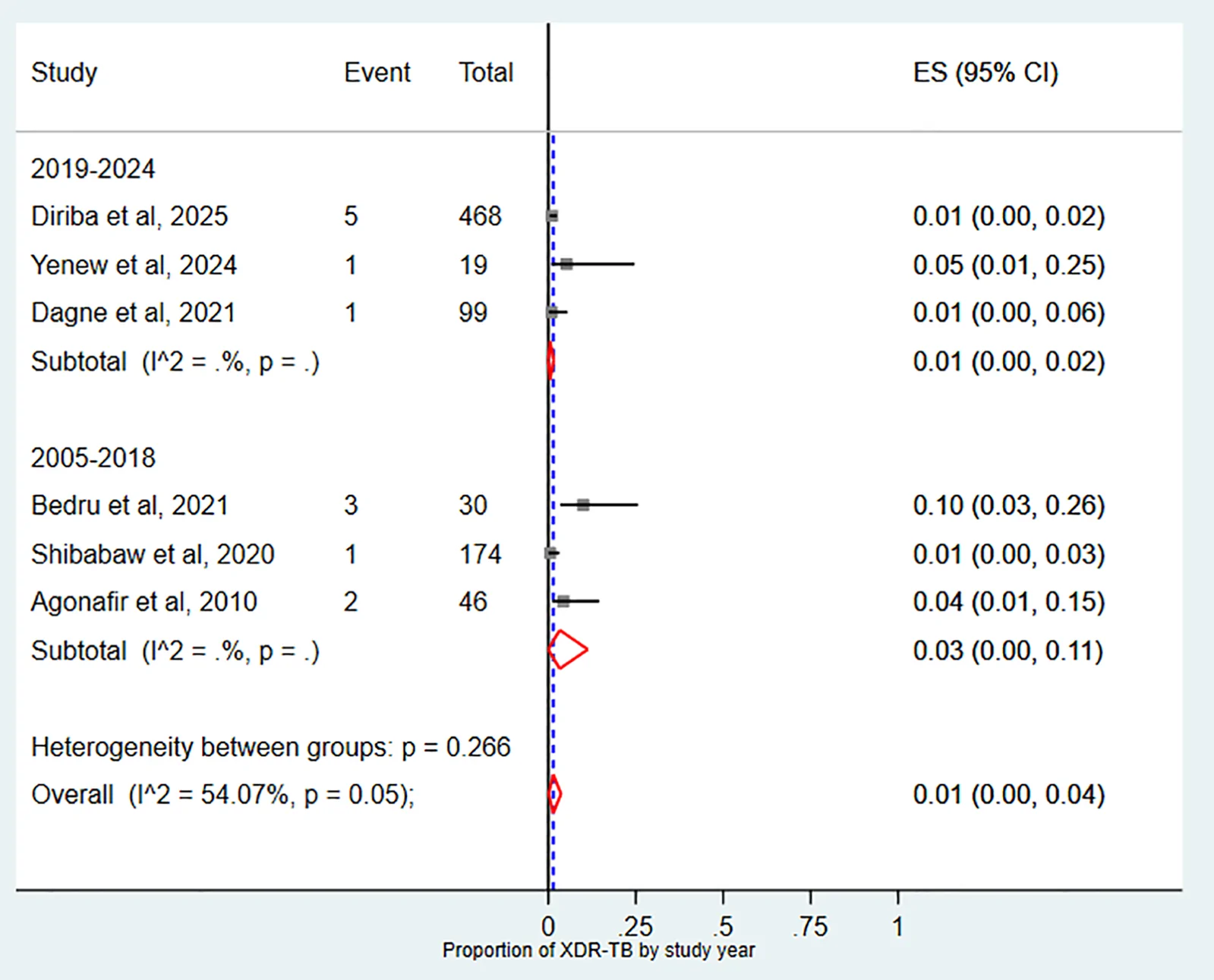
The forest plot showed XDR-TB subgroup analysis based on the study year.

**Fig 10:**
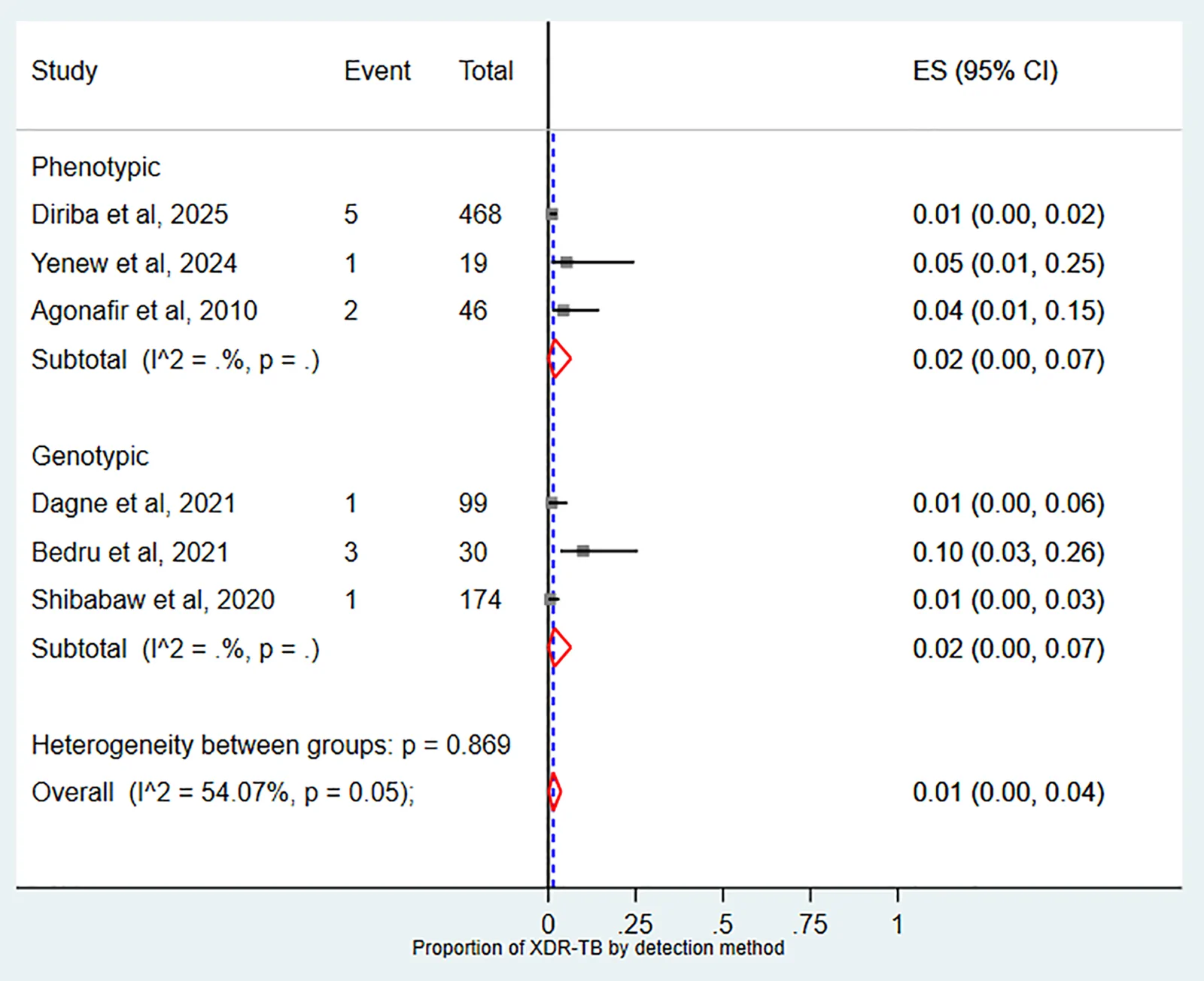
The forest plot showed the Pre-XDR-TB subgroup analysis based on the detection method.

### Publication bias

The presence of potential publication bias was statistically determined using Egger’s test. Egger’s test indicated non-significant publication bias for Pre-XDR-TB (p = 0.065), whereas there is a significant publication bias regarding XDR-TB (p = 0.024) (Table 2). Additionally, the distribution of the studies was depicted graphically using funnel plots (Figs 11 and 12).

**Table 2:** Publication bias using Egger’s test.

Category	Std_Eff.	Coef.	Std. Err.	T	p > t	[95% Conf. Interval]
Pre-XDR-TB	Slope	1.96	0.61	3.23	0.023	0.40-3.52
	Bias	1.2	0.51	2.35	0.065	-0.11-2.51
XDR-TB	Slope	0.16	0.30	0.55	0.613	-0.67-0.10
	Bias	1.27	0.36	3.54	0.024	0.28-2.27

**Fig 11:**
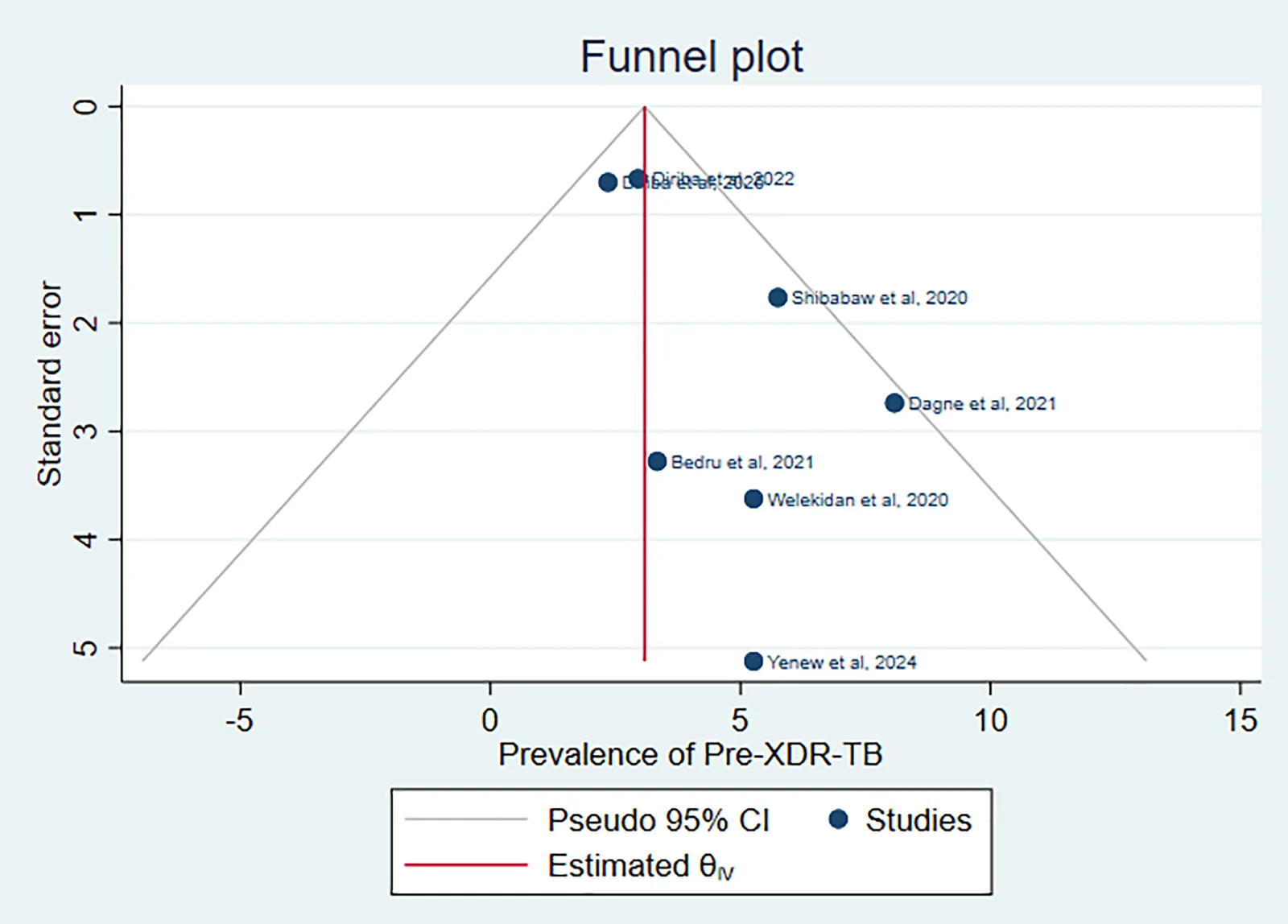
Funnel plot indicating publication bias for Pre-XDR-TB prevalence.

**Fig 12:**
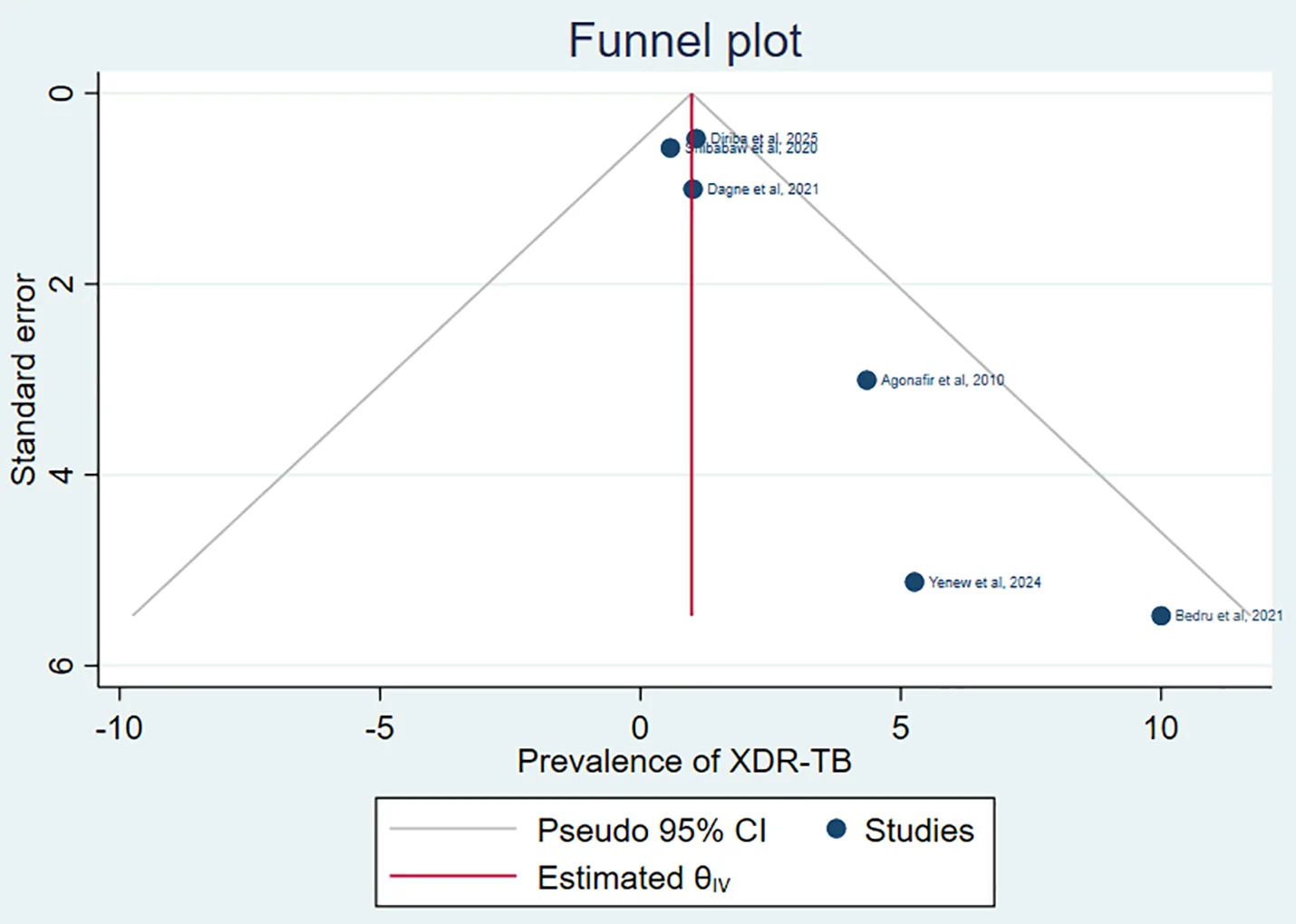
Funnel plot indicating publication bias for XDR-TB prevalence.

### Sensitivity analysis

Sensitivity analysis was performed to evaluate the heterogeneity between the studies reporting Pre-XDR-TB and XDR-TB prevalence. The step-by-step removal of each study was performed to determine the effect of each study on the pooled prevalence of Pre-XDR-TB or XDR-TB. The results showed that the omitted studies did not have a significant effect on the pooled effect sizes (Figs 13 and 14).

**Fig 13:**
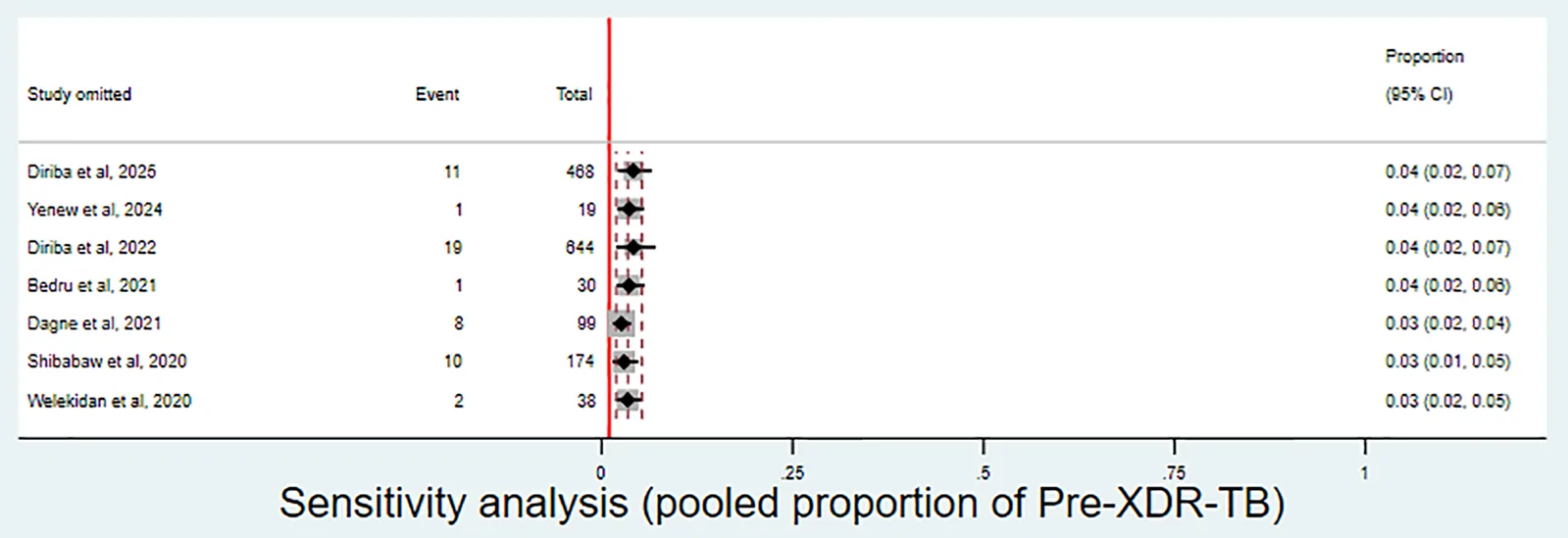
Sensitivity analysis for Pre-XDR-TB prevalence.

**Fig 14:**
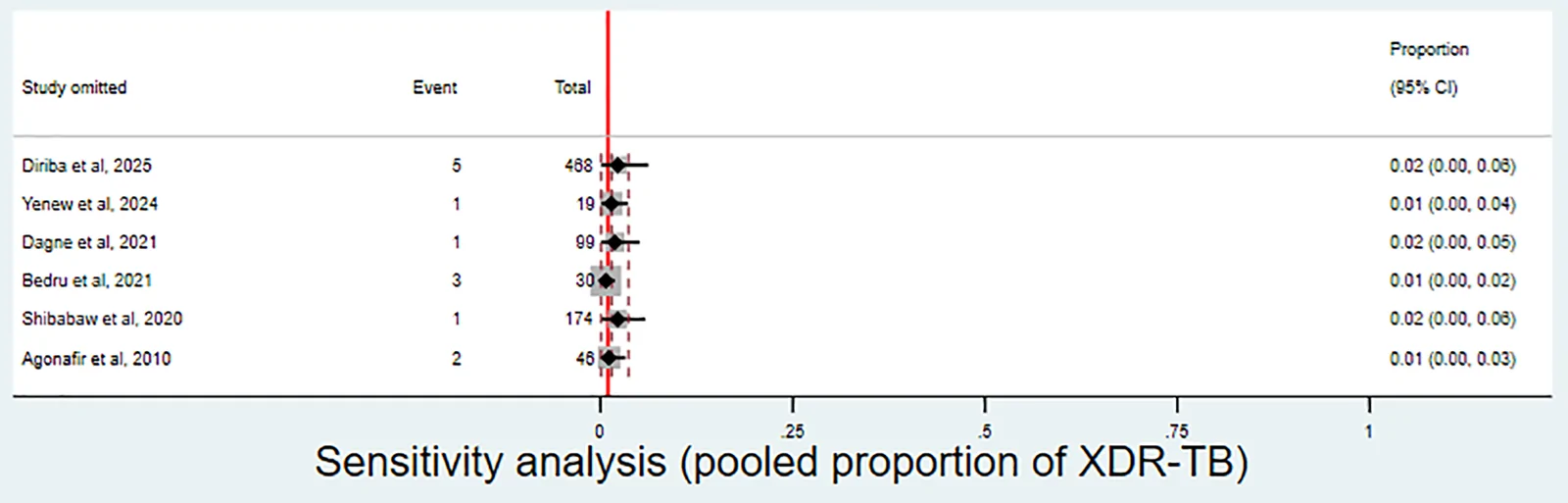
Sensitivity analysis for XDR-TB prevalence.

### Meta-regression

The meta-regression analysis revealed that sample size and publication year were not significant predictors of the pooled prevalence variation for either Pre-XDR-TB or XDR-TB (Table 3). This indicates that the prevalence rates reported across Ethiopia were consistent regardless of whether the study was conducted on a small or large scale, as well as year of publication.

**Table 3:** Meta-regression by sample size and publication year.

Category	Variables	Coef.	Std. Err.	t	p > t	[95% Conf. Interval]
Pre-XDR-TB	Sample size	-0.01	<0.001	-1.72	0.15	-0.01, < 0.001
	Publication year	-0.57	0.34	-1.68	0.15	-1.45, 0.30
XDR-TB	Sample size	< 0.001	0.005	0.03	0.98	-0.02, 0.02
	Publication year	-0.09	0.30	-0.29	0.79	-1.03, 0.85

## Discussion

This systematic review and meta-analysis aimed to determine the pooled prevalence of Pre-XDR and XDR-TB among patients diagnosed with MDR-TB in Ethiopia. In this study, the pooled prevalence of Pre-XDR and XDR-TB was 3% and 1%, respectively. A meta-analysis on 12,711 patients with MDR-TB from 22 countries reported the pooled proportion of Pre-XDR-TB; 26% and XDR-TB; 9% [22]. The pooled proportion of resistance to fluoroquinolones was 27% and second-line injectable drugs was 11%. Whereas the pooled resistance proportions to bedaquiline, clofazimine, delamanid, and linezolid were 5%, 4%, 5%, and 4%, respectively [22]. Based on the study from South Africa, 1247 TB cases were reported between 2019 and 2022, with 480 (38.5%) rifampicin-resistant TB, 63 (5.1%) Pre-XDR-TB, and 5 (0.4%) XDR-TB [23].

In Latin America and the Caribbean, the pooled prevalence of XDR-TB was 5%, with Cuba and Peru having higher pooled prevalences of 6% and 13%, respectively, while the pooled prevalence of Pre-XDR-TB was 10.0%, with Brazil and Peru having higher pooled prevalences of 16.0% and 13.0%, respectively [24]. Another study from Bangladesh reported 16.18% of Pre-XDR-TB cases, with 81.82% fluoroquinolone-resistant Pre-XDR-TB and 18.18% of second-line injectable agent-resistant Pre-XDR-TB [25]. Daniel et al. also found that the prevalence of Pre-XDR TB was 16.7%, with 20% and 80% of cases being resistant to kanamycin and ofloxacin, respectively [26]. A study reported that all Pre-XDR and XDR-TB isolates carried at least one mutation within the quinolone resistance-determining region of DNA gyrase [27]. According to the WHO global TB report in 2023, the estimated proportion of MDR/rifampicin-resistant-TB cases with Pre-XDR-TB was 19% [28]. This difference is related to study populations, epidemiological factors contributing to the evolution of drug resistance genes, and differences in laboratory detection methods and drug susceptibility testing of MTB. Considering recent molecular advancements in diagnostic methodologies, studies using the phenotypic detection method of drug resistance have been included in this meta-analysis, which may affect the overall pooled prevalence of Pre-XDR-TB and XDR-TB among MDR-TB cases in the country.

Since the WHO’s End TB strategy calls for a dramatic reduction in the TB burden and a high proportion of successfully treated cases, the increasing XDR-TB cases pose a huge challenge to this achievement. A meta-analysis showed that the global success rate of treating XDR-TB is 44.2%, which is lower than the WHO goal of a 75% success rate among individuals treated for TB [29]. A study in South Africa also reported a 10% treatment success outcome of XDR-TB patients [30]. Future research evaluating the treatment outcomes of XDR-TB with improved treatment regimens available may use these results as a guide. The interpretation of long-term trends in XDR-TB prevalence in Ethiopia must be approached with caution due to the 2021 revision of WHO definitions. Our analysis showed a prevalence of 3% in the 2005–2018 period compared to 1% in 2019–2024. This apparent decline may reflect older studies focused on resistance to injectable agents, while newer studies screen for Group A drugs. The differences in drug susceptibility testing panels across the two decades introduce a degree of non-comparability representing a limitation of any longitudinal meta-analysis in this field. In recent decades, the clinical threat of these drug-resistant MTB strains in Ethiopia is significantly explained by specific resistance patterns documented within the underlying primary studies, particularly regarding core Group A anti-TB drugs. Longitudinal data across the cohorts reveal that fluoroquinolone resistance has remained consistently between 3.0% and 3.4%, caused primarily by mutations in the quinolone resistance-determining region of the gyrA gene [16]. A recent surveillance from the 2022–2024 cohort reported resistance rates of 3.2% for fluoroquinolone and 1.71% for either bedaquiline or linezolid. The most alarming threat is the emergence of dual resistance strains, specifically combined fluoroquinolone and bedaquiline resistance in 0.85% and combined fluoroquinolone and linezolid resistance in 0.49% of MDR-TB cases [15]. Because the combination resistance is occurring predominantly among newly diagnosed, treatment-naive patients, these data signal that highly resilient resistant strains are actively circulating in the community.

This study found substantial heterogeneity across the study periods. Notably, the timeframe of the included studies (2005–2024) spans the pre-pandemic and COVID-19 pandemic eras. The pandemic significantly disrupted TB control programs in Ethiopia, leading to shifted laboratory priorities and reduced patient access to healthcare facilities. This may have resulted in an under-detection of Pre-XDR and XDR-TB cases during the 2020–2022 period, potentially contributing to the lower XDR-TB prevalence observed in more recent years (1% in 2019–2024 and 3% in 2005–2018). The diversion of Gene X-pert resources to COVID-19 testing likely created a surveillance gap that must be considered when interpreting these temporal trends. Regarding geographic variation, the higher prevalence of Pre-XDR-TB in certain regions, such as Addis Ababa, may be attributed to the presence of tertiary referral hospitals and specialized laboratories, such as the National Tuberculosis Reference Laboratory. These centers often manage the most complex, treatment-experienced MDR-TB cases from across the country, which naturally increases the likelihood of detecting advanced drug resistance. In contrast, more rural regions may report lower rates due to limited access to second-line drug susceptibility testing. Therefore, the observed regional differences likely reflect a combination of true biological variance and disparities in diagnostic infrastructure across Ethiopia.

Our meta-regression findings further support the robustness of the pooled estimates. The lack of a significant association between sample size and prevalence suggests that smaller studies, potentially biased their report to higher effect sizes, did not significantly affect our findings. 323 significantly distort our findings. This consistency across different study scales suggests that the observed burden of Pre-XDR and XDR-TB is a widespread challenge in Ethiopian MDR-TB treatment centers rather than a localized phenomenon observed only in smaller study reports. However, the relatively small number of included studies limits the statistical power of this regression. The Egger’s test result showed a potential publication bias for XDR-TB prevalence. The observed asymmetry in the funnel plot may also reflect the high level of heterogeneity and the rare cases of XDR-TB across the included studies. To guide the healthcare policy and improve patient treatment, comprehensive surveillance data on drug-resistant TB outcomes are essential, owing to the lack of laboratory facilities in most regions of Ethiopia. The current issue of Pre-XDR and XDR-TB in Ethiopia requires strengthening the national TB programs and continued efforts in TB prevention.

## Strengths and limitations

Despite providing a comprehensive synthesis of TB drug resistance in Ethiopia, this study has several important limitations. There is significant operational heterogeneity due to the evolution of the WHO XDR-TB definition in 2021. Older studies included in this review (2005–2018) primarily used second-line injectables, whereas newer studies (2019–2024) focused on Group A drugs (bedaquiline and linezolid). This shift, combined with variations in the coverage of drug susceptibility testing panels across different centers, complicates the comparability of results over time. The diagnostic methodology varied between studies, such as phenotypic and genotypic methods, which have different performance characteristics, particularly in their ability to detect specific mutations associated with Pre-XDR and XDR-TB, which may contribute to the observed heterogeneity. The small number of included studies limits the power of our meta-regression and Egger’s test. The limited regional coverage also means that our findings primarily represent the burden within specialized referral centers and may not be fully representative of the general MDR-TB population across all Ethiopian regions.

## Conclusion

This study reported a considerable prevalence of Pre-XDR (3%) and XDR-TB (1%) among MDR-TB cases in Ethiopia. Notably, recent studies showed a combined fluoroquinolone and bedaquiline/linezolid resistance in MDR-TB patients. Given the limited regional coverage and the small number of reported cases, further studies using advanced molecular techniques and new TB treatment regimens in each clinical setting should be conducted at the national level. Additionally, the presence of these highly resistant strains provides information to alert the public health, infection prevention, standardized diagnostic tools, and treatment options across the country.

## Supporting information

S1 Checklist Completed PRISMA 2020 checklist for systematic reviews. From: Page MJ, McKenzie JE, Bossuyt PM, Boutron I, Hoffmann TC, Mulrow CD, et al. The PRISMA 2020 statement: An updated guideline for reporting systematic reviews. BMJ 2021;372:n71. https://doi.org/10.1136/bmj.n71.
(DOCX)

S1 Table Differences from original review protocol. (DOCX)

S2 Table Database search terms. (DOCX)

S3 Table Quality assessment. (DOCX)
