# Supplementary material for: Pre-extensively and extensively drug-resistant tuberculosis prevalence among multidrug-resistant tuberculosis cases in Ethiopia: A systematic review and meta-analysis
Source: PLoS Negl Trop Dis. 2026 Sep 28;20(9):e0014744. doi: 10.1371/journal.pntd.0014744 (PMC13638038; doi:10.1371/journal.pntd.0014744)
Supplement: Supplementary file 2 [file pntd.0014744.s002.docx]

**S1 Table. Differences from the original review protocol**

| **Original review protocol** | **Difference** | **Rationale** |
| --- | --- | --- |
| - | We performed meta-analyses to estimate the pooled proportions of Pre-XDR and XDR-TB using the Freeman-Tukey double arcsine transformation. | To avoid negative pooled estimate values for rare events. |
