# Supplementary material for: Pre-extensively and extensively drug-resistant tuberculosis prevalence among multidrug-resistant tuberculosis cases in Ethiopia: A systematic review and meta-analysis
Source: PLoS Negl Trop Dis. 2026 Sep 28;20(9):e0014744. doi: 10.1371/journal.pntd.0014744 (PMC13638038; doi:10.1371/journal.pntd.0014744)
Supplement: Supplementary file 3 [file pntd.0014744.s003.docx]

| Databases | Search terms with Boolean operators |
| --- | --- |
| PubMed | (((((pre-extensively drug-resistant tuberculosis) OR (extensively drug-resistant tuberculosis)) OR (Pre-XDR-TB)) OR (XDR-TB)) AND ((multidrug-resistant tuberculosis) OR (MDR-TB))) AND (Ethiopia) |
| Scopus | TITLE-ABS-KEY(tuberculosis OR TB) AND TITLE-ABS-KEY("drug resistant" OR "multidrug-resistant" OR "MDR-TB" OR "pre-XDR" OR "XDR" OR "extensively drug-resistant") AND TITLE-ABS-KEY(prevalence OR epidemiology) AND TITLE-ABS-KEY(Ethiopia) |
| EMBASE | ('tuberculosis'/exp OR 'tuberculosis' AND ('drug resistance'/exp OR 'multidrug resistance' OR 'extensively drug resistance')) AND ('ethiopia'/exp OR 'ethiopia') AND ('prevalence'/exp OR 'epidemiology'/exp)  /exp searches for the term and all its more specific sub-terms in the tree. |
| Cochrane Library | (prevalence[MeSH Terms] OR epidemiolog*[tiab]) AND (tuberculosis, drug-resistant[MeSH Terms] OR "extensively drug-resistant tuberculosis"[tiab] OR "pre-extensively drug-resistant tuberculosis"[tiab]) AND (Ethiopia[MeSH Terms])  [MeSH Terms] limits the search to MeSH terms.  [tiab] limits the search to the title and abstract. |
| Google Scholar | "prevalence" "pre-extensively drug-resistant" "extensively drug-resistant" "multidrug-resistant tuberculosis" Ethiopia |

**S2 Table. Search terms used in different electronic databases.**
