# Supplementary material for: Pre-extensively and extensively drug-resistant tuberculosis prevalence among multidrug-resistant tuberculosis cases in Ethiopia: A systematic review and meta-analysis
Source: PLoS Negl Trop Dis. 2026 Sep 28;20(9):e0014744. doi: 10.1371/journal.pntd.0014744 (PMC13638038; doi:10.1371/journal.pntd.0014744)
Supplement: Supplementary file 4 [file pntd.0014744.s004.docx]

**S3 Table. Quality assessment of the studies included in systematic review and meta-analysis on the prevalence of Pre-XDR-TB and XDR-TB among MDR-TB cases in Ethiopia.**

| Author, year of publication | Q1 | Q2 | Q3 | Q4 | Q5 | Q6 | Q7 | Q8 | Q9 | Total score (9) |
| --- | --- | --- | --- | --- | --- | --- | --- | --- | --- | --- |
| Diriba et al, 2025 | Y | Y | Y | Y | Y | Y | Y | Y | Y | 9 |
| Diriba et al, 2022 | Y | Y | Y | Y | Y | Y | Y | Y | Y | 9 |
| Dagne et al, 2021 | Y | Y | Y | Y | Y | Y | Y | Y | Y | 9 |
| Yenew et al, 2024 | Y | Y | N | Y | Y | Y | Y | Y | Y | 8 |
| Welekidan et al, 2020 | Y | Y | N | Y | Y | Y | Y | Y | NA | 7 |
| Bedru et al, 2021 | Y | Y | N | Y | Y | Y | Y | Y | Y | 8 |
| Shibabaw et al, 2020 | Y | Y | Y | Y | Y | Y | Y | Y | Y | 9 |
| Agonafir et al, 2010 | N | Y | Y | Y | Y | Y | Y | Y | Y | 8 |

**Key:Y**= Yes; **N**= Not reported, **NA**=Not appropriate

**Question codes:**

1. Was the sample frame appropriate to address the target population?

2. Were study participants sampled in an appropriate way?

3. Was the sample size adequate?

4. Were the study subjects and the setting described in detail?

5. Wasthedataanalysisconducted with sufficient coverageoftheidentified sample?

6. Were valid methods used for the identification of the condition?

7. Was the condition measured in a standard, reliable way for all participants?

8. Was there appropriate statistical analysis?

9. Was the response rate adequate, and if not, was the low response rate managed appropriately?
